# Study of Structure and Permeability Relationship of Flavonoids in Caco-2 Cells

**DOI:** 10.3390/nu9121301

**Published:** 2017-11-29

**Authors:** Yajing Fang, Weiwei Cao, Mengmeng Xia, Siyi Pan, Xiaoyun Xu

**Affiliations:** Key Laboratory of Environment Correlative Dietology, Ministry of Education, Huazhong Agricultural University, Wuhan 430070, China; fyj@webmail.hzau.edu.cn (Y.F.); weiweicao@webmail.hzau.edu.cn (W.C.); xiamengmeng@webmail.hzau.edu.cn (M.X.); Pansiyi@mail.hzau.edu.cn (S.P.)

**Keywords:** Caco-2, flavonoids, intestinal absorption, permeability, QSPR

## Abstract

Flavonoids exhibit a broad range of biological activities. However, poor absorption of some flavonoids is a major limitation for use of flavonoids as nutraceuticals. To investigate the structure requirements for flavonoids intestinal absorption, transepithelial transport and cellular accumulation (CA) of 30 flavonoids were determined using the Caco-2 cell monolayer. The bilateral permeation of five types of flavonoids followed the order: flavanones ≥ isoflavones > flavones ≥ chalcones > flavonols. The concentration of flavonoids accumulated in cells did not correlate with cell penetration since the correlation coefficient between the apparent permeability coefficient (*P*_app_) and their corresponding CA was poor (*R*^2^ < 0.3). Most flavonoids exhibited a ratio of 0.8–1.5 for *P*_app A to B_/*P*_app B to A_, suggesting passive diffusion pathways. However, luteolin, morin and taxifolin may involve the efflux mechanisms. The quantitative structure-permeability relationship (QSPR) study demonstrated that the intestinal absorption of flavonoids can be related to atomic charges on carbon 3′ (*Q*_C3′_), molecule surface area (*S*log*P_V*3), balance between the center of mass and position of hydrophobic region (*vsurf_ID*1) and solvation energy of flavonoids (*E_sol*). These results provide useful information for initially screening of flavonoids with high intestinal absorption.

## 1. Introduction

Flavonoids which are produced by plants as secondary plant metabolites are one of the most widely distributed polyphenols. Structurally, flavonoids are derived from a parent nucleus, a diphenylpropane (C6-C3-C6) skeleton. Flavonoids are mainly divided into eight subclasses depending on the structure difference on C ring, including flavones, flavonols, flavanones, flavanonols, isoflavones, flavan-3-ols, flavan-3, 4-diols (flavandiol) and anthocyanins [1]. Flavonoids exhibit health benefits due to their various biological activities including antioxidant, anti-cancer [2], antimicrobial [3], anti-inflammation [4] and others. However, poor oral bioavailability of some flavonoids, caused by low epithelial transport and extensive metabolism, is a major limitation for use of flavonoids as nutraceuticals. Low bioavailability has been associated with flavonoid interactions at various stages of the digestion, absorption and distribution process, which are strongly affected by their molecular structures. Hence, an increase in absorption of flavonoids is one way to improve their oral bioavailability. Previous studies have shown that the molecular structures play an important role on the absorption of flavonoids, including the degree or position of substitution of hydroxyl and alkyl group [5], methoxyl group [6], prenyl group [7], and glycosidic group [8]. 

Numerous structure permeability relationship (SPR) studies of flavonoids have been investigated by comparing the difference of permeability/absorption data and structure of analogues. However, the application of SPR study has been limited due to lack of prediction for untested compounds. The quantitative structure activity relationship (QSAR) for flavonoids is gaining interest by quantitatively correlating the molecular structures or properties with variation in biological activity. But, few QSPR studies have been performed to investigate the intestinal absorption of flavonoids [9] and the absorption mechanisms for flavonoids are not well established. 

Caco-2 monolayer model is widely used [10,11] to estimate and predict the intestinal permeability of various flavonoids [5,12], because it is derived from human colonic adenocarcinoma and shares many morphological (e.g., microvilli) and functional properties with mature enterocytes. Caco-2 cells also exhibit a well-differentiated brush border on their apical surfaces and express many typical transporters and enzymes found in the small intestine [13,14]. An excellent correlation was found between the oral absorption of flavonoids in human and the apparent permeability (*P*_app_) in Caco-2 monolayer model when the *P*_app_ is more than 10^−6^ cm/s [15].

In this study, 30 flavonoids were investigated for their intestinal permeability and cellular accumulation (CA) by using the Caco-2 monolayer model. First, the tested flavonoids with diverse structures including flavones, flavonols, flavanones, flavanonols, isoflavones and chalcones were studied to obtain their SPR. To observe the effect of structure of flavonoids on their intestinal absorption, a 2D-QSPR model was developed using the *P*_app_ values from apical to basolateral side (*P*_app A to B_) as the dependent variable and molecular descriptors as independent variables. The chemical structures of the 30 flavonoids tested are presented in Table 1.

## 2. Materials and Methods

### 2.1. Materials and Methods

Human colon adenocarcinoma cell line Caco-2 (ATCC #HTB-37) was purchased from American Type Culture Collection (ATCC) (Rockville, MD, USA). 3-(4,5-dimethylthiazol-2-yl)-2,5-diphenyltetrazolium bromide (MTT) was purchased from Gen-View Scientific Inc. (Calimesa, CA, USA). Compound **1** and **2** were purchased from BBT INC. (Tianjin, China) (purity > 98%). Compound **8** was purchased from Shanghai Tauto Biotech Co., Ltd. (Shanghai, China) (purity > 98%). Compound **16** (purity > 95%) and Lucifer yellow carbohydrazide (CH) were purchased from Sigma-Aldrich (St. Louis, MO, USA). The remaining flavonoid compounds were purchased from Aladdin Chemistry Co., Ltd. (Shanghai, China) (purity > 98%). The flavonoids were dissolved in stock with a concentration of 100 mM anddiluted to a final concentration of 0.1% (*v*/*v*) dimethyl sulfoxide. Fetal bovine serum (FBS) was obtained from Gibco Laboratories (Life Technologies Inc., Grand Island, NY, USA). Minimum essential medium (MEM) and non-essential amino acids (NEAA) were purchased from Hyclone (Logan, UT, USA). Millicell hanging cell culture inserts of 12 wells (PET, 12 mm, pore size 0.4 μm) were purchased from Millipore (Boston, MA, USA) and 12-well plates were purchased from Corning Costar (Cambridge, MA, USA). All other chemicals were of analytical grade.

### 2.2. Cell Viability Assay

The cytotoxicity of flavonoids and colchicine was evaluated by MTT assay. The cells were grown in 96-well plates at a density of 1 × 10^4^ cells/well. After incubation with 40 μM flavonoid for 24 h, the cells were further incubated with an MTT solution (0.5 mg/mL) for 4 h at 37 °C. Finally, after removal of the supernatant, 150 μL dimethyl sulphoxide was added to each well to dissolve crystallized MTT. Absorbance was read at 570 nm with a multiskan spectrum microplate reader (Thermo Labsystems, Waltham, MA, USA). The percentage cell viability relative to that of the control cells was used as the cytotoxicity measure. 

### 2.3. The Permeability of Flavonoids in Caco-2 Cell Monolayers

Caco-2 cells were cultured in MEM supplemented with 10% FBS, 1% NEAA, penicillin (100 U/mL) and streptomycin (100 μg/mL) in an atmosphere of 5% CO_2_ and 90% relative humidity at 37 °C. All cells used in this study were between passages 35 and 45 [16]. 

Caco-2 cells were seeded at a density of about 8 × 10^4^ cells/cm^2^ on a 12 wells Millicell hanging insert and left to grow for 19–21 days to reach confluence. Culture medium was replaced every other day for 14 days and daily thereafter. The integrity and transport ability of the Caco-2 cells monolayer were examined by measuring the trans-epithelial electrical resistance (TEER) with a Millicell voltammeter (Millicell ERS-2, Merck Millipore, Billerica, MA, USA) and running standard assays using Lucifer yellow CH as paracellular flux marker. Only cell monolayers with a TEER above 600 Ω·cm^2^ and *P*_app_ of Lucifer yellow CH flux less than 0.5 × 10^−6^ cm/s were used for the transport assays. Differentiation of Caco-2 cells was checked after 4 and 12 days by determining the activity of alkaline phosphatase with an assay kit (Nanjing Jiancheng Bioengineering Institute, Nanjing, China) and after 21 days transmission electron microscopy was performed on Caco-2 monolayer to evaluate differentiation [17].

The Caco-2 cell monolayer was washed with D-Hank’s buffer (pH 7.4). Then, the flavonoid (40 μM) was added to either the apical (AP, 0.4 mL) or basolateral (BL, 1.95 mL) side, while the receiving chamber contained the corresponding volume of D-Hank’s buffer and incubated for 1 h at 37 °C. Solutions from both AP and BL sides of Caco-2 cell monolayer were collected and immediately frozen, lyophilized and preserved at −80 °C for high performance liquid chromatography (HPLC) analysis. *P*_app_ was calculated from Equation (1), where Δ*Q*/Δ*t* is the rate of the flavonoid on the accepting chamber (μM/s), A is the surface area of the hanging insert (cm^2^) and *C*_0_ is the initial concentration of tested flavonoid in donating chamber (μM/mL).

(1) $$P_{\mathrm{app}}=\frac{\Delta Q/\Delta t}{AC_{0}}$$

### 2.4. The CA of Flavonoids in Caco-2 Cell Monolayer

After the collection of samples from both AP and BL sides, the hanging insert was rinsed twice with ice-cold D-Hank’s buffer to stop further transport, and lysed with 0.1% Triton X-100 in D-Hank’s buffer then placed in an ultrasonic bath for 15 min. Cell lysates were collected and centrifuged at 7000× *g* for 10 min. Supernatants were analyzed by BCA kit (Dingguochangsheng Biotechnology, Beijing, China) for protein content. After protein content assay, supernatants were immediately frozen, lyophilized and preserved at −80 °C. The flavonoid concentration in lysis solution was determined by HPLC and normalized with cellular protein content.

### 2.5. HPLC Assays of Flavonoids

To determine both the permeability and CA of flavonoids in Caco-2 cells, the lyophilized samples were dissolved in running 120 to 200 μL methanol, and centrifuged at 7000× *g* for 5 min. Aliquot of 20 μL of the supernatant solution was used for assay using Waters e2695 HPLC system (Milford, MA, USA) fitted with an Amethyst C18-H column (250 mm × 4.6 mm i.d., 5 μm; Sepax, Newark, DE, USA). The mobile phase consisted of A (0.01 M phosphoric acid (H_3_PO_4_))/B (acetonitrile) with 1.0 mL/min flow rate. The assay was performed using the following gradient: 0–5 min: 95% A; 5–10 min: 95–50% A; 10–20 min: 50% A; 20–22 min: 50–95% A; 22–30 min: 95% A. Elution peaks were monitored with a diode-array detector at the wavelength of maximum absorption of each flavonoid (200–400 nm). Peak area measurement was used to obtain standard calibration curves to determine concentration of each flavonoid.

### 2.6. 2D-QSPR Study

The structures of 30 flavonoids were drawn by Chembiodraw ultra 12.0 and energy minimization was performed by Sybyl X-2.0 (Tripos Inc., St. Louis, MO, USA). The geometric structures of these compounds were optimized using the density functional methods (DFT) calculations at the level of Becke’s-parameter hybrid functional (B3LYP) and polarized basis sets 6-31G (*d*, *p*). The electronic and topological descriptors and molecular properties were calculated by Gaussian 09 program package, Sybyl X-2.0 and molecular operating environment (MOE) 2009. 

The p*P*_app A to B_ (−log (*P*_app A to B_)) was used as the dependent variable and descriptors were used as independent variables in the 2D-QSPR study. Twenty-two compounds were chosen by random as the training set to build the QSPR model and the remaining six compounds (**9**, **12**, **14**, **20**, **27** and **30**) were used as the test set. Correlation analysis was performed by stepwise linear regression and bivariate methods to select the molecular descriptors affecting the permeability of flavonoids. Collinear descriptors, with the absolute value of inter-correlation coefficient (*R*) higher than 0.7, were omitted [18]. The 2D-QSPR model was achieved using the partial least squares (PLS) algorithms with default parameters in Sybyl X-2.0. The resulting model was validated by using cross-validation (leave-one-out) procedures. Cross-validation coefficient (*Q*^2^) should be more than 0.5 for a reliable model [3]. The root-mean-square error (RMSE) for the training set was measured as shown in Equation (2) to evaluate the predictability of the developed model [19].

(2) $$\mathrm{RMSE}=\sqrt{\frac{\sum_{i=1}^{n}{\left(Y_{\mathrm{iexp}.}-Y_{\mathrm{ipred}.}\right)}^{2}}{n}}$$

### 2.7. Statistical Analysis

Statistical differences were determined by student’s *t* test on SPSS 16.0 and *p* values less than 0.05 and 0.01 were considered significant and very significant, respectively. 

## 3. Results

### 3.1. Cell Viability

Cell viability was evaluated in Caco-2 cells treated with 40 μM flavonoid. As shown in Table 2, there is no significant difference (*p* < 0.05) between the Caco-2 cell viability of the control and each flavonoid. These results indicated that the flavonoids at the tested concentration showed no cytotoxicity to Caco-2 cells. 

### 3.2. Transport of Flavonoids

Based on Caco-2 cell viability, the same nontoxic concentration (40 μM) was used for all flavonoids in the transport studies to minimize error resulting from the concentration-dependent effect shown by some flavonoids. 

The flavonoids permeated across the membrane to the acceptor compartments in both the apical to basolateral (A to B) and the basolateral to apical (B to A) assays. The results of bidirectional transport are summarized in Table 3. The flavonoid with the highest *P*_app_ is **26** (a isoflavonoid) for both *P*_app A to B_ and *P*_app B to A_ with a value of (33.90 ± 3.55) × 10^−6^ cm/s and (42.19 ± 3.11) × 10^−6^ cm/s, respectively, followed by **19**, **21** and **27** with *P*_app_ more than 30 × 10^−6^ cm/s. Other isoflavonoid aglycones (**23**, **25**, and **28**) also showed good permeability with *P*_app_ more than 10 × 10^−6^ cm/s. Flavanone aglycones (**18** and **22**) also showed good intestinal absorption with high *P*_app_ more than 20 × 10^−6^ cm/s. These results indicate that isoflavonoids and flavanones show higher permeability than other flavonoids. Flavone aglycones display modest good intestinal absorption with *P*_app_ greater than 6 × 10^−6^ cm/s while the low permeability (<2 × 10^−6^ cm/s) of compound **4** results from the easily oxidized pyrogallic group. The flavonols with *P*_app_ values less than 10 × 10^−6^ cm/s were poorly absorbed. The permeability of flavonols was lower than flavones by comparing **5** versus **10**, **6** versus **12** and **7** versus **9**, respectively. The hydroxyl group at position 3 may be unfavorable for flavonoids to pass through the cell membrane, leading to low permeability for flavonols from both apical and basolateral sides. Flavonoid glycosides (**8**, **16**, **17**, **20**, **24** and **30**) showed low *P*_app_ values (<6 × 10^−6^ cm/s), suggesting that the presence of glycosidic group is also unfavorable for the absorption of flavonoids. 

Most of the flavonoids exhibited a ratio*_p_* ranging 0.8–1.5 (Table 3), suggesting that transport of these flavonoids is by passive diffusion. However, compounds **5**, **11** and **22** with ratio*_p_* >2.0, exhibit efflux in Caco-2 monolayer. The absorption for compounds **4** and **25** (ratio*_p_* < 0.5) is good, and also indicates higher absorption from apical to basolateral side. 

### 3.3. CA of Flavonoids in Caco-2 Cell Monolayers

The CA in cells from apical to basolateral side (CA _A to B_) and basolateral to apical side (CA _B to A_) of flavonoids after transport are summarized in Table 4. The CA from both sides (CA _A to B_ and CA _B to A_) of flavonoids **14**, **16**, **17**, **22**, and **24** were not detected. The other two flavonoid glycosides **8** and **30** also showed very low CA (<0.26 μmol/g), suggesting that both the accumulation of flavonoid glycosides and their transport (low *P*_app_) were poor. This may result from the low hydrophobicity of these flavonoids, which makes penetration through the phospholipid bilayer cell membrane difficult. Compound **14**, with an unstable pyrogallic group, is easily autoxidized, resulting in low concentration both in cells (not detected) and transport chamber (*P*_app_ < 0.5 × 10^−6^ cm/s). Compounds **19**, **21, 26** and **27** showed low CA values (<5 μmol/g) compared with their highest *P*_app_ (>30 × 10^−6^ cm/s). Compounds **12** and **28** showed highest CA (>6 μmol/g) with modest *P*_app_. These results suggest that the concentration of flavonoid aglycones accumulated in cells is not correlated with cell penetration. This is confirmed by the poor correlation coefficient (*R*^2^ < 0.3) between the *P*_app_ and their corresponding CA.

The ratio_c_ was within the range of 0.09–2.77, as shown in Table 4. The ratio_c_ values for most of the compounds (except compounds **2**, **13**, **15** and **25**) were more than 1.00, implying greater accumulation of flavonoids in the basolateral to apical direction. The CA _A to B_ exhibited significant (*p* < 0.05 or *p* < 0.01) differences from the CA _B to A_ for most of the flavones (except compound **2** and **3**), isoflavones (except compound **23**) and all of the flavonols. These results indicated that flavonoids accumulated in Caco-2 cells to a different extent during bilateral transport.

### 3.4. Stability of Flavonoids

In our initial study (data not shown), we found that methanol is a good solvent for the dissolution of flavonoids, even better than the mobile phase B (acetonitrile) used in HPLC assay. Hence, we evaluated the stability of flavonoids in different solvents with methanol as a standard solvent. The relative stability was expressed as the relative appearance of flavonoids in different solvents. Each flavonoid (20 μM) was dissolved in two different solvents, D-Hank’s and methanol. The relative concentration of flavonoid in D-Hank’s was calculated by Equation (3) with results summarized in Table 5. The relative concentration was more than 50% for most of the tested flavonoids, suggesting that most flavonoids are stable in D-Hank’s buffer. However, the values of **4**, **13** and **14** were lower than 40%, suggesting that these flavonoids are unstable in D-Hank’s buffer. That could explain the low experimental *P*_app_ of these three flavonoids.

Relative concentration in D-Hank’s (%) = Peak area in D-Hank’s/Peak area in methanol × 100%(3)

The recovery of the flavonoid glycosides during the transport assays was determined by mass balance and was measured as total amount of the flavonoids on both sides of the insert and in the Caco-2 cells. The recovery rate and relative standard deviation (RSD) values of seven flavonoid glycosides are shown in Table 5. Recoveries were all greater than 60% for these flavonoid glycosides, indicating that flavonoid glycosides are stable during the transport assay, and the low permeability for these flavonoids is not due to their stability.

### 3.5. Construction and Validation of the QSPR Model

To evaluate the relationship between intestinal absorption and structure of flavonoids, a QSPR model was constructed with p*P*_app A to B_ as the dependent variable and physio-chemical properties descriptors as independent variables. Stepwise regression analysis was used for variable selection to obtain the best equation. At the beginning of modeling, compounds **4** and **13** with high residuals (experimental p*P*_app A to B_ minus predicted p*P*_app A to B_ > 0.600) were omitted as outliers. Several factors may contribute to the outlier status of these two compounds, including low permeability and structure uniqueness. For compound **4**, a specific pyrogallic group makes it unstable, as illustrated by its low relative concentration (34.12%) in D-Hank’s, and it has been shown to be metabolically susceptible [20]. For compound **13**, the low relative concentration (7.25%) and highest predictive residual (1.011) led to the outlier of this compound. The best QSPR equation was obtained from 22 compounds in training set as shown in Equation (4).

(4) $$pP_{\mathrm{app}\;A\;\mathrm{to}\;B}=4.715+1.358Q_{C3\prime }+0.059E\_sol+0.020S\log P\_V3+0.056vsurf\_ID1$$

*R*^2^ = 0.881, *R*^2^_adj_ = 0.875, *Q*^2^ = 0.810, *F* = 44.509, *p* < 0.01, RMSE = 0.141, *R*^2^_pred_ = 0.842, *R*^2^_adj-pred_ = 0.802, SEE = 0.156

**Figure 1:**
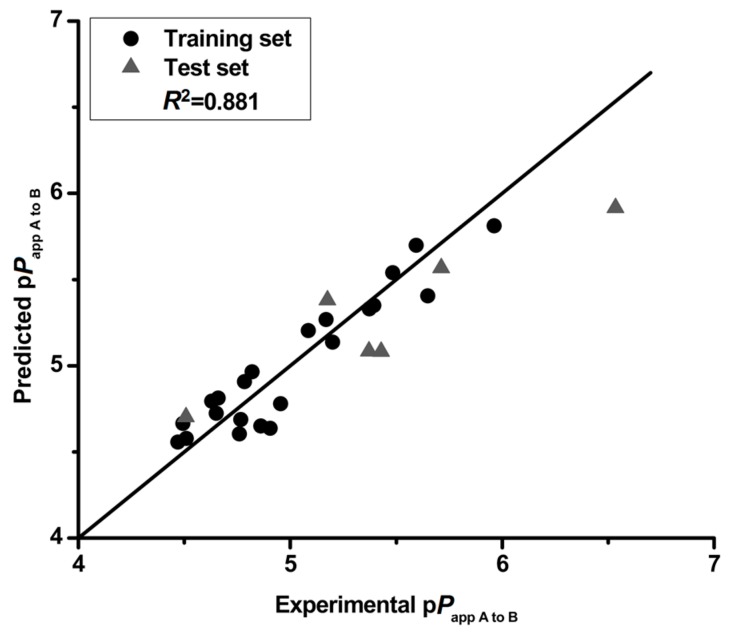
The experimental p*P*_app A to B_ versus predicted p*P*_app A to B_.

The correlation matrix between p*P*_app A to B_ and the respective molecular properties are shown in Table 6. The correlation matrix showed that the properties are independent, demonstrating that the model in this study is robust. Moreover, the QSPR model was validated by cross-validation (leave-one-out) and a high cross-validated coefficient (*Q*^2^ = 0.810) was achieved. The p*P*_app A to B_ both in training and test set is predicted by this QSPR model with a particularly high degree of accuracy (Figure 1). The predicted deviations of the tested compounds (except **14** and **15**) both in training and test set were lower than 0.5 as shown in Table 7. The QSPR is considered of high predictive ability with a high value (0.841) of square of predictive correlation coefficient (*R*^2^_pred_) for the test set. In addition, the low RMSE value (0.242) also confirmed the accuracy of the developed model. These results illustrate that the QSPR model is robust and predictive, and could be used to predict the intestinal absorption of flavonoids.

### 3.6. QSPR Study

In this study, four descriptors, namely *Q*_C3′_, *E_sol*, *S*log*P_V*3 and *vsurf_ID*1, were selected to build the QSPR model. This model showed that higher values for the descriptors results in a decrease in intestinal absorption of flavonoids. The significant Pearson coefficients between *Q*_C3′_ or *S*log*P_V*3 and p*P*_app A to B_ suggests that these two descriptors play major roles in the transport of flavonoids. The electronic properties of flavonoids are important for their absorption. The molecule charge of a flavonoid is electro-neutral, while, the electronic delocalization for each atom in a molecule varies with the number and position of substituents. The value of *Q*_C3′_ for each flavonoid is shown in Table 7, representing the atomic charges of carbon 3′ of the molecule. By comparing the *Q*_C3′_ and structure difference for tested flavonoids (Table 1 and Table 7), the π–π-conjugation on B ring makes this ring in a stable electron distribution and carbon 3′ with small positive atomic charges (0.011) when without substitution at this position (**1**, **3**, **4**, **7** and **9**). The atomic charges increased to higher positive atomic charges (approximately 0.300) with the presence of hydroxyl or methoxyl group (**5**, **10**, **14**, **15**, **16**, **17**, **22** and **30**). The oxygen of hydroxyl/methoxyl group withdraws the electron(s) to the oxygen atom side leading to a loss of carbon 3′ electron(s) resulting in positive atomic charges for carbon 3′. This can be confirmed by comparing compound **27** and **29**, with same *S*log*P_V*3 and similar *vsurf_ID*1, although **29** has a lower *E_sol* which favors absorption, **27** with a lower *Q*_C3′_ showed better absorption. The hydroxylation or methoxylation of adjacent carbon 4′ (**2**, **6**, **8**, **11**, **19**, **20**, **21**, **24**, **25**, **26**, **27**, **28** and **29**) results in carbon 4′ having positive charges, and carbon 3′ having negative charges. Similarly, it can be presumed that substitution on carbon 2′ will also increase absorption. This is verified when evaluating flavonoids **11** and **12**. Flavonoid **11** having negative charges on carbon 3′, with substitution both on carbon 2′ and carbon 4′ showed better absorption than **12** with substitution only on carbon 4′. These results suggest that the substitution by an electronegative group (such as a hydroxyl group) on carbon 3′ is unfavorable for flavonoid absorption while the substitution on adjacent carbon (2′ and 4′) favors absorption. 

*S*log*P_VSA* descriptors aim to capture hydrophilic and hydrophobic effects in the receptor and the atomic contribution to logP was calculated by the Wildman and Crippen SlogP model [19]. *S*log*P_VSA*3 (*S*log*P_V*3) represent the Van der Vaals surface area of the atoms contributing to the logP (o/w) of the molecule in the range (0, 0.1). High *S*log*P_V*3 increases the molecular surface area, resulting in low absorption of flavonoids. For flavonols, the substitution of a hydroxyl group on position 3 increases *S*log*P_V*3 to 25.386. Flavonoid glycosides (**8**, **17**, **20**, **24** and **30**), with substitution of a glycosidic group also showed an increase in *S*log*P_V*3 (>20.93). Flavonoid **8**, having two glycosidic groups at two different positions (6 and 8), showed the highest *S*log*P_V*3 value (41.853). These results suggest that the substitution of 3-OH or glycosidic group(s) is unfavorable for the absorption of flavonoids.

The vsurf descriptors relating to structure connectivity and conformation are similar to the VolSurf descriptors [21]. *vsurf_ID*1 is a hydrophobic integy moment vector pointing from the center of mass to the center of a hydrophobic region. The descriptor represents the unbalance between the center of mass of a molecule and the position of the hydrophobic regions around it at a −0.2 kcal/mol energy level. A high integy moment indicates that the hydrophobic regions are clearly concentrated in only one part of the molecular surface. A small integy moment indicates that the barycenter is close to the center of the molecule. Compounds **25** and **26** displayed same *S*log*P_V*3 and *Q*_C3′_ and similar *E_sol* however **26** with lower *vsurf_ID*1 showed better absorption because **25** had an external methoxyl group on position 6. The flavonoid glycosides (**8**, **16**, **17**, **20**, **24** and **30**) showed high integy moment (>2.7) resulting from bulky glycosidic group(s), which could aggravate the unbalance between the barycenter and hydrophobic region position. Flavonoids (**5**, **11**, **21**, **23** and **26**) showed small integy moment (<0.5). The numbers of substitution on A and B ring are the same for these flavonoids so that the barycenter is close to the center of the molecule. For compound **7**, two hydroxyl groups (position 5 and 7) are far away from the center of mass leading to a high integy moment (8.594) which is similar to compound **2** also having a high integy moment (4.097). In summary, when the distribution of substituents is equal between A and B ring, the balance between the center of hydrophobic region and the center of mass will increase, resulting in high absorption of flavonoids. However, the substitution of a balky group (such as a glycosidic group) decreases the balance between the center of hydrophobic region and the center of mass, thereby decreasing the absorption of flavonoids.

*E_sol* is a 3D molecular descriptor, representing the solvation energy of flavonoids in the solvation process. The higher the energy is, the lower the resulting absorption. By comparing compounds **20** and **24**, with same *S*log*P_V*3 and *Q*_C3′_ and similar *vsurf_ID*1, compound **20**, with lower *E_sol* showed better absorption. Similarly, with lower *E_sol*, compound **27** had higher absorption than **25**. For flavonoids **18** and **19,** having similar *S*log*P_V*3 and *vsurf_ID*1, **19** with much higher *E_sol*, showed better absorption than **18** despite having a slightly lower *Q*_C3′_. These results indicate that *E_sol* is an important parameter related to the absorption of flavonoids.

## 4. Discussion

The bilateral permeation across the Caco-2 monolayer of selected five types of flavonoids (Table 1) followed the order: flavanones ≥ isoflavones > flavones ≥ chalcones > flavonols, which is consistent with previous results reported for the bioavailability of flavonoids (isoflavones > flavanols > flavanones > flavonols > anthocyanins) [22]. The permeability for most of tested flavonoids in this study is similar with the literature results [5,16,23,24] since their bidirectional permeability was in the same order of magnitude. The bilateral permeation of flavonoids followed the order: **12** > **10** > **15**, consistent with a previous study [16]. The permeability of Lucifer yellow CH is also consistent with that reported in a previous study [16]. Our results indicate that the Caco-2 monolayer in this study is well suited to represent flavonoid transport and the data are valid. 

Flavonoid glycoside **20** naringin, (*P*_app_ < 6 × 10^−6^ cm/s) showed much lower permeability than **19** naringenin, (*P*_app_ > 32.13 × 10^−6^ cm/s). The other flavonoid glycosides tested also showed low permeability (*P*_app_ < 6 × 10^−6^ cm/s). These results are in agreement with studies that concluded that glycosides are poorly absorbed compounds [23,25]. Poor lipid solubility and the presence of multiple hydroxyl groups are reported reasons contributing to the poor absorption of flavonoid glycosides [25]. However, the *P*_app_ value reported is lower (<1 × 10^−6^ cm/s) in previous studies [23,26] than in our study. The discrepancy could be associated in part to the poor membrane permeability of these glycosides, as well as to different experimental conditions used. The CA of glycosides in this study are low (<0.3 μmol/g) or not detected which is agreement with a literature result [23]. Although the absorption of flavonoid glycosides in vitro is poor or not detected, it was confirmed that the absorption of quercetin glycosides in a human study with ileostomy subjects was higher than quercetin, implying that absorption of glycosides in the small intestinal is possible [27].

Flavonols showed lower permeability than corresponding flavones when comparing **5** versus **10, 6** versus **12** and **7** versus **9**, suggesting 3-OH is unfavorable for flavonoid permeability. In addition, flavonoids **4**, **9**, **10**, **13**, **14** and **15**, sharing three or more hydroxyl groups showed low permeability. The *P*_app_ values increased adversely with the number of hydroxyl groups in a sub class of flavonoids as reported previously [22]. Possible explanation include excess free hydroxyl groups are easily autoxidized, especially the presence of a pyrogallic group which can easily bind hydrogen bonds with its aqueous environment [28] and rapid conjugation by glucuronidation and sulfation [29].

An influential predictive model for intestinal absorption is the “Lipinski rule-of 5”, which resulted from the analysis of the World Drug Index [30]. Compounds that are likely absorbable through the intestines contain no more than 5 H-bond donors, 10 H-bond acceptors, have a molecular weight of <500 Da and log*P* (lipophilicity index) <5. According to this rule, flavonoids, with higher numbers of hydroxyl, or glycosidic moieties are less likely to be absorbed through the intestines which are in agreement with our results. In contrast, methoxylated flavonoids lose their H-bond acceptor/donor properties and have higher log*P* values, which make them highly absorbable. Compound **2** containing methoxyl groups showed high permeability, consistent with the literature that concluded methylation greatly improved intestinal absorption [31]. For flavonoids with only one methoxyl group, it is not certain that absorption will increase. Our study found that methoxylation increases the absorption when comparing compound **3** and **7**, but decreases absorption by comparing **10** versus **15**, **25** versus **26** and **27** versus **28**, respectively. 

Most flavonoids exhibited a ratio of *P*_app A to B_/*P*_app B to A_ from 0.66 to 1.50, indicating that they permeate across the Caco-2 monolayer via passive diffusion. For compounds **5**, **11** and **22**, however, the hypothesis of active transport should not be excluded since the ratio*_p_* is higher than 2 [15]. For **22**, the ratio*_p_* (4.13), is much higher than 2, suggesting an efflux mechanism which was also concluded in a previous study [32]. The transport mechanism of compound **10** with a high ratio*_p_* (1.84), warrants further study since this compound has been reported to be pumped back by breast cancer resistance protein (BCRP) in the form of glucuronidated metabolites [33]. Chrysin (compound **7**) has been shown to exhibit efflux in Caco-2 monolayer [34]. In this study, efflux of chrysin was not observed probably due to the design of HPLC assays for which only parental compound were determined with standard of chrysin. The relative concentration of chrysin in D-Hank’s is less than 50%, indicating that it is not stable in the buffer solution. Besides the rule of ratio*_p_* [15], it is more concise and popular to use inhibitor of transporters to distinguish flavonoid absorption, especially, for poorly absorbed flavonoids. Further study is needed to focus on the efflux transport for flavonoids since it has been reported that passive diffusion and carrier-mediated efflux are known to be the two major pathways for a molecule to permeate across the intestinal epithelium [35].

Due to the wide range and variability in structure within each group, it is difficult to generalize the absorption of flavonoids based only on structure. Physio-chemical properties need to be taken into account to ascertain structure relationship to permeability. In this study, QSPR model was built to construct a mathematical relationship between the molecular structural descriptors and permeability (*P*_app A to B_). QSPR devoted to flavonoid absorption is limited in the literature. 2D and 3D QSPR models were constructed to explain and predict intestinal absorption of flavonoids using *P*_app A to B_ in Caco-2 cells [9]. However, the data consisted of the log of the *P*_app_ (transformed to *P*_app_ × 10^−7^), which omitted the part of 10^−7^ was not precise in the previous study [9]. The data were transformed to p*P*_app_ (−log) in this study is more acceptable than the previous study [9]. Moreover, the atom-type electrotopological state (E-state) and weighted holistic invariant molecular (WHIM) descriptors used to develop QSPR model were reported to be cumbersome to interpret [9]. Therefore, no structure and permeability relationship was discussed with those models and those models only lies on their utility in screening of emerging flavonoids [9]. In addition, the 3D model (comparative molecular similarity index analysis, CoMSIA) reported in the literature was not suitable since CoMSIA is usually used for a target receptor (such as enzyme) [36] but not for permeability properties. Because the permeability of flavonoids can be influenced by several factors, such as pumped out by efflux transporters [24], interaction with membrane [5], stability, solubility and hydrophobicity of the compounds [37]. 

In QSPR analysis, hydrophobic, electronic and steric are the three major descriptors that must be included in analysis [38]. In our study, the descriptors used to build QSPR model were derived from more than one molecular modeling package, Gaussian 09, SYBYL X-2.0 and MOE 2009 including physical, electrostatic, topological, hydrophobic, and energetic descriptors. Physical 3D structure of flavonoids is indeed a determining factor for intestinal absorption since our QSPR model captured three 3D descriptors among four descriptors. *Q*_C3′_ used in our study representing the atomic charge on carbon 3′ is easily to interpret when comparing with those descriptors in previous study [9]. The substitution (methoxylation or hydroxylation) on carbon 3′ or adjacent positions affects *Q*_C3′_, and also affects flavonoid absorption. Hydroxylation or methoxylation at carbon 3′ will decrease absorption of a flavonoid while substitution on adjacent carbon 2′ or 4′ can increase the absorption. This type of descriptor has been successfully used in generating a quantitative structure-activity relationship (QSAR) model to predict antibacterial capacity against *E. coli* for flavonoids [3]. *S*log*P_V3* was proven to have a negative effect on absorption, which is consistent with a study that reported that an increase in polar surface area (*PSA*) was unfavorable for absorption [39]. Such descriptor has been successfully used in QSAR modeling of HIV-1 reverse transcriptase inhibition by benzoxazinones [36] and inhibition of potassium channel blockers [40]. It was found in this study that the presence of 3-OH or glycosidic group decreases flavonoid absorption according to *S*log*P_V*3. *vsurf_ID*1 representing the balance of the molecule, indicates favorable absorption when the substitution is equal on A and B ring. Descriptors of this type were used to develop a model for discriminating P-gp substrates based on Caco-2 efflux ratio values [41]. *E_sol* describes the process of solubilisation of flavonoids and is utilized in the prediction of solubility [42]. The increasing of the energy will decrease the solubility thereby decrease flavonoids absorption. These descriptors generate a direct link between the molecular parameters of the model to the actual structural properties that govern the permeability and they are easy to interpret. 

## 5. Conclusions

Oral bioavailability remains one of the most critical issues for flavonoids in nutrition and health. In this study, 30 flavonoids were tested for transepithelial permeability in Caco-2 cell monolayer. QSPR model was built to predict intestinal absorption of flavonoids by using structural characteristics and the model showed good predictive power. Four descriptors, *Q*_C3′_, *S*log*P_V*3, *vsurf_ID*1 and *E_sol*, were found to relate to flavonoid absorption. Substitution of a negative group (such as -OH or -OCH_3_) on carbon 3′ decreases the flavonoid absorption while substitution on adjacent carbon 2′ or/and carbon 4′ increases the absorption. The presence of 3-OH or glycosidic group decrease the absorption of flavonoids. The equal substitution on A ring and B ring is also favorable for absorption. The results of this study are important in ascertaining the structural features important to flavonoid intestinal absorption.

## Figures and Tables

**Table 1:** The chemical structures of 30 flavonoids.

No.	Flavonoids	Core Structure	R_3_	R_5_	R_6_	R_7_	R_8_	R_2′_	R_3′_	R_4′_	R_5′_
**1**	Flavone	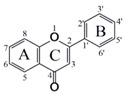	H	H	H	H	H	H	H	H	H
**2**	Tangertin		H	OMe	OMe	OMe	OMe	H	H	OMe	H
**3**	Wogonin		H	OH	H	OH	OMe	H	H	H	H
**4**	Baicalein		H	OH	OH	OH	H	H	H	H	H
**5**	Luteolin		H	OH	H	OH	H	H	OH	OH	H
**6**	Apigenin		H	OH	H	OH	H	H	H	OH	H
**7**	Chrysin		H	OH	H	OH	H	H	H	H	H
**8**	Schaftoside		H	OH	Cglc	OH	Carb	H	H	OH	H
**9**	Galangin		OH	OH	H	OH	H	H	H	H	H
**10**	Quercetin		OH	OH	H	OH	H	H	OH	OH	H
**11**	Morin		OH	OH	H	OH	H	OH	H	OH	H
**12**	Kaempferol		OH	OH	H	OH	H	H	H	OH	H
**13**	Kaempferide		OH	OH	H	OH	H	H	H	OMe	H
**14**	Myricetin		OH	OH	H	OH	H	H	OH	OH	OH
**15**	Isorhamnetin		OH	OH	H	OH	H	H	OMe	OH	H
**16**	Quercitrin		Orha	OH	H	OH	H	H	OH	OH	H
**17**	Rutin		ORG	OH	H	OH	H	H	OH	OH	H
**18**	Hesperetin	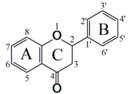	H	OH	H	OH	H	H	OH	OMe	H
**19**	Naringenin		H	OH	H	OH	H	H	H	OH	H
**20**	Naringin		H	OH	H	ONG	H	H	H	OH	H
**21**	Liquiritigenin		H	H	H	OH	H	H	H	OH	H
**22**	Taxifolin		OH	OH	H	OH	H	H	OH	OH	H
**23**	Formononetin	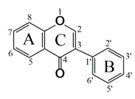	-	H	OH	H	H	H	OMe	H	H
**24**	Puerarin		-	H	H	OH	Cglc	H	H	OH	H
**25**	Glycitein		-	H	OMe	OH	H	H	H	OH	H
**26**	Daidzein		-	H	H	OH	H	H	H	OH	H
**27**	Genistein		-	OH	H	OH	H	H	H	OH	H
**28**	Biochanin A		-	OH	H	OH	H	H	H	OMe	H
**29**	Isoliquiritigenin	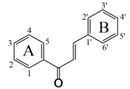	OH	OH	-	-	-	H	H	OH	H
**30**	Neohesperidin dihydrochalcone	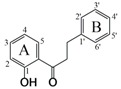	NG	OH	-	-	-	H	OH	OMe	H

Cglc: -C-glucopyranosyl; Carb: -*O*-(α-l-Arabinopyranosyl); Orha: -*O*-α-l-rhamnopyranosyl; RG: -(6-*O*-(6-deoxy-α-l-mannopyranosyl)-β-d-glucopyranosyloxy); NG: -(2-*O*-(6-deoxy-α-l-mannopyranosyl)-β-d-glucopyranosyl).

**Table 2:** The cell viability of 30 flavonoids in Caco-2 cells.

No.	Cell Viability (%)	No.	Cell Viability (%)	No.	Cell Viability (%)
**1**	96.32 ± 9.79 *^a^*	**12**	100.22 ± 4.53 *^a^*	**23**	94.60 ± 3.60 *^a^*
**2**	97.52 ± 3.53 *^a^*	**13**	101.77 ± 6.15 *^a^*	**24**	105.49 ± 6.13 *^a^*
**3**	97.68 ± 8.39 *^a^*	**14**	100.79 ± 3.15 *^a^*	**25**	98.35 ± 4.60 *^a^*
**4**	96.72 ± 6.31 *^a^*	**15**	93.64 ± 1.22 *^a^*	**26**	105.02 ± 4.56 *^a^*
**5**	100.66 ± 4.19 *^a^*	**16**	98.38 ± 3.86 *^a^*	**27**	103.27 ± 3.37 *^a^*
**6**	101.09 ± 4.56 *^a^*	**17**	105.78 ± 5.48 *^a^*	**28**	105.78 ± 6.44 *^a^*
**7**	103.02 ± 4.94 *^a^*	**18**	100.12 ± 2.70 *^a^*	**29**	99.18 ± 7.94 *^a^*
**8**	103.40 ± 4.77 *^a^*	**19**	95.60 ± 3.47 *^a^*	**30**	102.04 ± 6.35 *^a^*
**9**	106.20 ± 7.79 *^a^*	**20**	99.06 ± 4.92 *^a^*	**C**	100.00 ± 4.38 *^a^*
**10**	102.16 ± 5.19 *^a^*	**21**	100.74 ± 4.27 *^a^*		
**11**	101.86 ± 3.62 *^a^*	**22**	99.48 ± 2.00 *^a^*		

C: Control. Results are presented as means ± Standard Deviation (SD), *n* = 5. Statistical comparisons were made using the one way ANOVA and a Duncan test. *^a^*: Same letters mean there is no significance between the tested flavonoid and control in Caco-2 cells.

**Table 3:** Apparent permeability coefficients (*P*_app_) of flavonoids in Caco-2 monolayer.

No.	*P*_app A to B_ × 10^−6^ cm/s	*P*_app B to A_ × 10^−6^ cm/s	Ratio*_p_*	No.	*P*_app A to B_ × 10^−6^ cm/s	*P*_app B to A_ × 10^−6^ cm/s	Ratio*_p_*
**1**	22.35 ± 1.37	21.73 ± 1.21	0.97	**16**	4.24 ± 1.25	5.96 ± 1.20	1.41
**2**	21.86 ± 0.585	23.94 ± 0.45	1.10	**17**	4.04 ± 0.07	4.28 ± 0.44	1.06
**3**	16.43 ± 1.20	18.07 ± 1.62	1.10	**18**	23.50 ± 0.85	27.92 ± 2.76	1.19
**4**	1.95 ± 0.53	0.71 ± 0.21	0.37	**19**	32.13 ± 2.98	35.34 ± 1.16	1.10
**5**	10.10 ± 7.33	21.45 ± 2.12	2.12	**20**	3.73 ± 0.73	5.09 ± 1.55	1.36
**6**	17.12 ± 1.72	16.92 ± 1.02	0.99	**21**	30.97 ± 0.80	36.96 ± 1.97	1.19
**7**	6.78 ± 0.12	8.41 ± 0.62	1.24	**22**	6.32 ± 1.16	26.08 ± 2.08	4.13
**8**	3.28 ± 0.32	2.81 ± 0.24	0.86	**23**	17.42 ± 1.78	18.31 ± 1.83	1.05
**9**	1.94 ± 0.41	1.75 ± 0.44	0.90	**24**	2.25 ± 0.96	2.70 ± 0.41	1.20
**10**	2.55 ± 1.45	4.68 ± 0.41	1.84	**25**	12.45 ± 0.91	6.23 ± 0.46	0.50
**11**	8.23 ± 0.87	21.58 ± 0.32	2.62	**26**	33.90 ± 3.55	42.19 ± 3.11	1.24
**12**	6.68 ± 0.93	5.92 ± 0.45	0.89	**27**	31.07 ± 2.13	30.48 ± 2.13	0.98
**13**	0.35 ± 0.06	0.23 ± 0.03	0.66	**28**	11.11 ± 0.51	10.70 ± 2.98	0.96
**14**	0.29 ± 0.01	0.44 ± 0.01	1.50	**29**	13.76 ± 0.73	20.04 ± 1.29	1.46
**15**	1.09 ± 0.02	0.90 ± 0.42	0.83	**30**	4.25 ± 0.53	5.08 ± 1.08	1.20

*P*_app A to B_: Transport of a flavonoid from apical to basolateral side; *P*_app B to A_: Transport of a flavonoid from basolateral to apical side; Ratio_*P*_: the ratio of *P*_app B to A_ to *P*_app A to B_. Data are means ± SD (*n* = 3–6). *P*_app_ value of Lucifer yellow carbohydrazide (CH) was about (3.58 ± 0.12) × 10^−7^ cm/s. The incubation time was 60 min.

**Table 4:** The cellular accumulation (CA) of flavonoids in Caco-2 monolayer.

No.	CA _A to B_ (μmol/g)	CA _B to A_ (μmol/g)	Ratio_c_	No.	CA _A to B_ (μmol/g)	CA _B to A_ (μmol/g)	Ratio_c_
**1**	1.932 ± 0.051	3.183 ± 0.504 *^a^*	1.65	**16**	ND	ND	-
**2**	2.706 ± 0.174	2.423 ± 0.543	0.90	**17**	ND	ND	-
**3**	1.038 ± 0.212	1.586 ± 0.045	1.53	**18**	1.293 ± 0.086	1.644 ± 0.300	1.27
**4**	1.182 ± 0.341	3.272 ± 0.445 *^a^*	2.77	**19**	1.935 ± 0.305	4.436 ± 0.299 *^b^*	2.29
**5**	3.876 ± 0.741	8.639 ± 1.275 *^b^*	2.23	**20**	0.058 ± 0.013	0.068 ± 0.011	1.17
**6**	4.415 ± 0.467	7.610 ± 0.694 *^b^*	1.72	**21**	0.635 ± 0.146	1.234 ± 0.249 *^a^*	1.94
**7**	3.902 ± 0.251	8.828 ± 0.213 *^b^*	2.26	**22**	ND	ND	-
**8**	0.024 ± 0.002	0.044 ± 0.007	1.83	**23**	3.819 ± 1.320	4.842 ± 0.424	1.27
**9**	3.780 ± 0.711	9.286 ± 0.859 *^b^*	2.46	**24**	ND	ND	-
**10**	NT	NT	-	**25**	1.821 ± 0.774 *^a^*	0.158 ± 0.021	0.09
**11**	0.129 ± 0.008 *^b^*	0.060 ± 0.011	0.464	**26**	0.500 ± 0.104	1.236 ± 0.117 *^b^*	2.47
**12**	8.368 ± 1.039	11.887 ± 1.247 *^a^*	1.42	**27**	1.415 ± 0.276	2.777 ± 0.220 *^b^*	1.96
**13**	2.746 ± 0.478 *^b^*	0.496 ± 0.249	0.18	**28**	6.766 ± 1.583	14.087 ± 1.287 *^b^*	2.08
**14**	ND	ND	-	**29**	4.393 ± 0.790	8.804 ± 1.576 *^a^*	2.00
**15**	0.332 ± 0.066 *^a^*	0.076 ± 0.048	0.23	**30**	0.096 ± 0.010	0.251 ± 0.012	2.61

CA _A to B_: the content of the flavonoids that accumulated in the cell monolayer after transport from apical to basolateral side. CA _B to A_: the content of the flavonoids that accumulated in the cell monolayer after transport from basolateral to apical side. Ratio_c_: the ratio of CA _B to A_ to CA _A to B_. Data are means ± SD, *n* = 3. *^a^*: means *p* < 0.05, *^b^*: means *p* < 0.01 in *t* test. ND: not detected. NT: not tested.

**Table 5:** Flavonoid stability.

**Relative Concentration of Flavonoids**					
**No.**	D-Hank’s/methanol (%)	**No.**	D-Hank’s/methanol (%)	**No.**	D-Hank’s/methanol (%)
**1**	60.10	**10**	52.10	**19**	113.93
**2**	12.09	**11**	79.06	**20**	64.99
**3**	51.64	**12**	98.60	**21**	71.02
**4**	34.12	**13**	7.25	**24**	30.31
**5**	87.51	**14**	ND	**25**	85.60
**6**	41.76	**15**	90.67	**26**	74.83
**7**	47.63	**16**	116.67	**27**	88.16
**8**	72.75	**17**	66.82	**28**	81.33
**9**	105.96	**18**	115.83	**29**	131.85
**Recovery of 7 Flavonoid Glycosides**					
**No.**	Recovery (B to A, %)	RSD (%)	Recovery (A to B, %)	RSD (%)	
**8**	61.83 ± 0.72	1.16	72.31 ± 18.76	25.96	
**11**	90.12 ± 1.39	1.56	81.45 ± 6.70	8.22	
**16**	76.77 ± 9.36	12.19	90.69 ± 8.85	9.76	
**17**	96.95 ± 1.67	1.72	98.18 ± 4.74	4.84	
**20**	82.88 ± 10.99	13.26	93.17 ± 7.62	8.18	
**24**	85.25 ± 0.60	0.70	87.42 ± 3.31	3.79	
**30**	79.50 ± 1.24	1.58	78.66 ± 1.30	1.65	

B to A: Recovery of transport of a flavonoid from basolateral to apical side. A to B: Recovery of transport of a flavonoid from apical to basolateral side. RSD: relative standard deviation. ND: not detected.

**Table 6:** The Pearson Correlation between p*P*_app A to B_ and related descriptors.

	p*P*_app A to B_	*Q*_C3′_	*E_sol*	*S*log*P_V3*	*vsurf_ID*1
p*P*_app A to B_	1.000	0.576 **	0.045	0.738 **	0.407
*Q*_C3′_		1.000	−0.273	0.223	0.214
*E_sol*			1.000	−0.103	−0.363
*S*log*P_V3*				1.000	−0.148
*vsurf_ID*1					1.000

**: Correlation is significant at the 0.01 level.

**Table 7:** Calculated results using the QSPR model.

	No.	p*P*_app A to B_ (Exper)	p*P*_app A to B_ (Pred)	*E_sol*	*S*log*P_V3*	*Q*_C3′_	*vsurf_ID*1	Residuals
Training set	**1**	4.651	4.756	−0.789	0	0.010	0.736	−0.105
	**2**	4.660	4.947	−1.802	0	−0.019	4.097	−0.287
	**3**	4.784	4.803	1.526	0	0.011	1.561	−0.019
	**5**	4.820	4.663	−3.225	0	0.305	0.474	0.157
	**6**	4.766	4.751	−0.893	0	−0.007	0.636	0.015
	**7**	5.169	5.197	0.986	0	0.011	8.594	−0.028
	**8**	5.484	5.709	−2.750	41.853	−0.004	2.774	−0.225
	**10**	5.594	5.278	0.378	25.386	0.301	0.822	0.316
	**11**	5.085	5.243	0.104	25.386	−0.033	0.359	−0.158
	**15**	5.962	5.322	1.610	25.386	0.322	1.034	0.640
	**16**	5.373	5.041	−2.100	0	0.272	6.626	0.332
	**17**	5.394	5.363	−10.491	20.927	0.276	8.232	0.031
	**18**	4.629	4.750	1.351	0	−0.028	0.685	−0.121
	**19**	4.493	4.754	−1.043	0	−0.018	0.669	−0.261
	**21**	4.509	4.731	−2.117	0	−0.019	0.228	−0.222
	**22**	5.199	4.755	−0.789	0	0.302	1.064	0.444
	**23**	4.759	4.735	−1.910	0	−0.012	0.334	0.024
	**24**	5.648	5.370	1.320	20.927	−0.034	4.292	0.278
	**25**	4.905	4.749	−1.314	0	−0.023	0.553	0.156
	**26**	4.470	4.730	−2.317	0	−0.023	0.190	−0.260
	**28**	4.954	4.753	0.600	0	−0.006	0.690	0.201
	**29**	4.861	4.765	−1.601	0	−0.013	0.861	0.096
Test set	**9**	5.712	5.347	3.668	25.386	0.005	2.195	0.365
	**12**	5.175	5.254	2.066	25.386	0.005	0.548	−0.079
	**14**	6.535	5.376	3.979	25.386	0.295	1.045	1.159
	**20**	5.429	5.514	−6.238	20.927	−0.034	6.492	−0.085
	**27**	4.508	4.745	−0.205	0	−0.022	0.527	−0.237
	**30**	5.372	5.062	−10.370	20.926	0.291	3.024	0.310

QSPR: quantitative structure-permeability relationship.

## Abbreviations

CA	cellular accumulation
CoMSIA	comparative molecular similarity index analysis
FBS	fetal bovine serum
HPLC	high performance liquid chromatography
MEM	minimum essential medium
NEAA	non-essential amino acids
*P*_app_	the apparent permeability coefficient
PLS	partial least squares
SPR	structure-permeability relationship
QSPR	quantitative structure-permeability relationship
QSAR	quantitative structure-activity relationship
RMSE	the root-mean-square error
TEER	trans-epithelial electrical resistance
